# Supplementary material for: Disentangling kinetics from thermodynamics in heterogeneous colloidal systems
Source: Nat Commun. 2023 Feb 4;14:607. doi: 10.1038/s41467-023-36292-8 (PMC9899263; doi:10.1038/s41467-023-36292-8)
Supplement: Supplementary file 1 — Supplementary Information [file 41467_2023_36292_MOESM1_ESM.pdf]

Supplementary Information for:

Disentangling kinetics from thermodynamics in heterogeneous colloidal systems

Hamed Almohammadi[1], Sandra Martinek[1], Ye Yuan[1], Peter Fischer[1], Raffaele Mezzenga[1,2*]

[1]Department of Health Sciences and Technology, ETH Zurich, Zurich, Switzerland

[2]Department of Materials, ETH Zurich, Zurich, Switzerland

*Correspondence to: raffaele.mezzenga@hest.ethz.ch

Supplementary Fig. 1

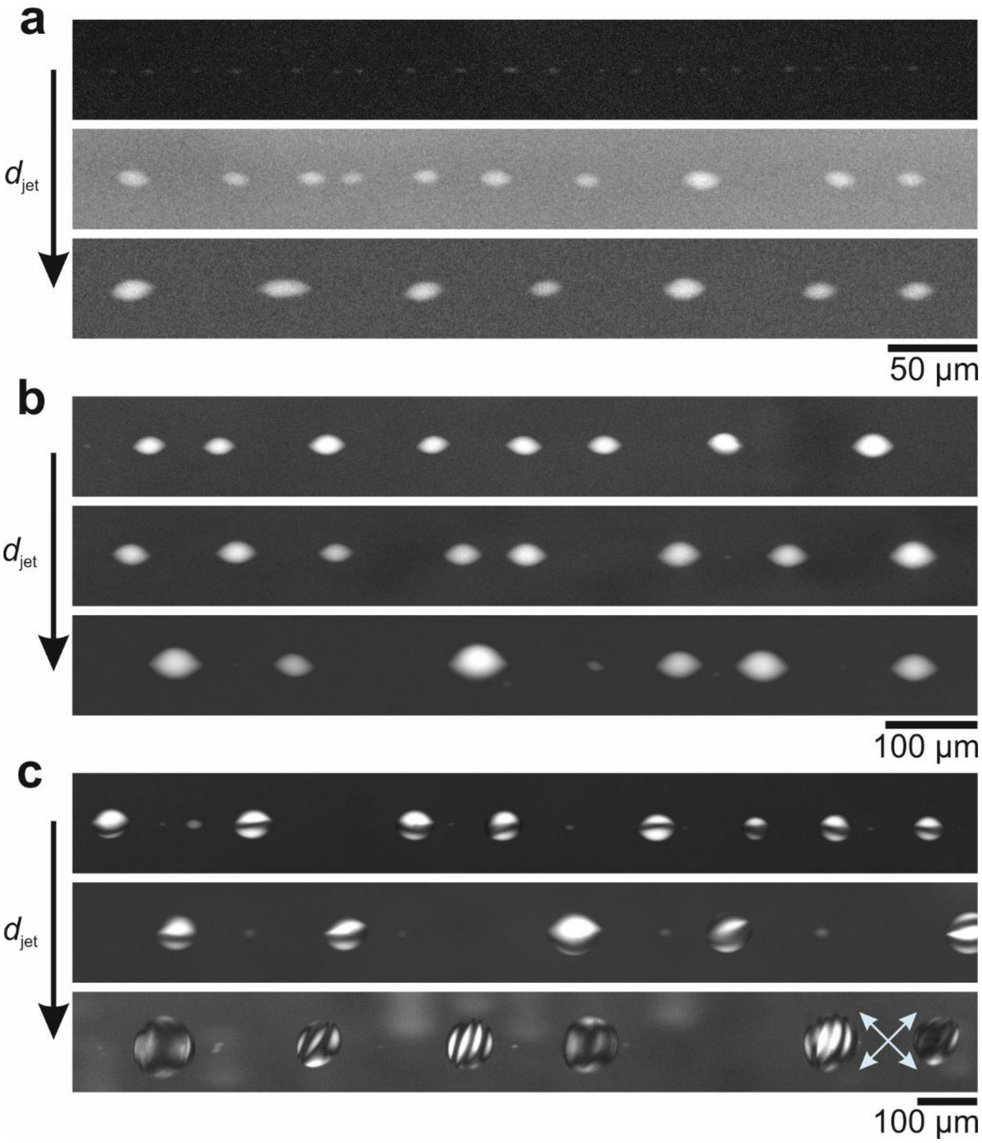

Supplementary Fig. 1 | Formation of nematic and cholesteric amyloid fibrils tactoids with various sizes, shapes, and internal structures. Homogenous (**a**), bipolar (**b**), and cholesteric (**c**) tactoids with different volumes are formed by extruding nematic jets with various diameters, which are achieved by adjusting q_{in} and q_{out}. All tactoids are oriented with their long axes in the flow direction, except cholesteric tactoids with four or higher number of bands that are oriented almost perpendicular to the flow direction. The crossed arrows represent the crossed polarizers.

Supplementary Fig. 2

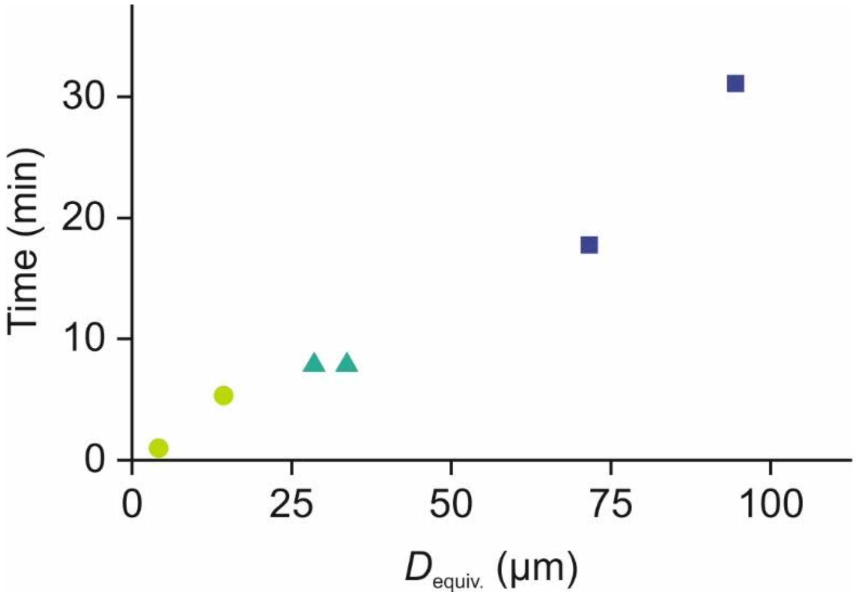

Supplementary Fig. 2 | *Induction time* of the liquid crystalline tactoids formed by extruding the Onsager branches. The filled circle, triangle, and square symbols denote homogenous, bipolar, and cholesteric tactoids, respectively. The results show that the *induction time* increases with an increase in the volume of the tactoids and, importantly, it is in the order of the minutes. *Induction time* is defined as the time elapsed from the moment the jet is extruded till when the tactoids reach their equilibrium states (see Fig. 2a).

Supplementary Fig. 3

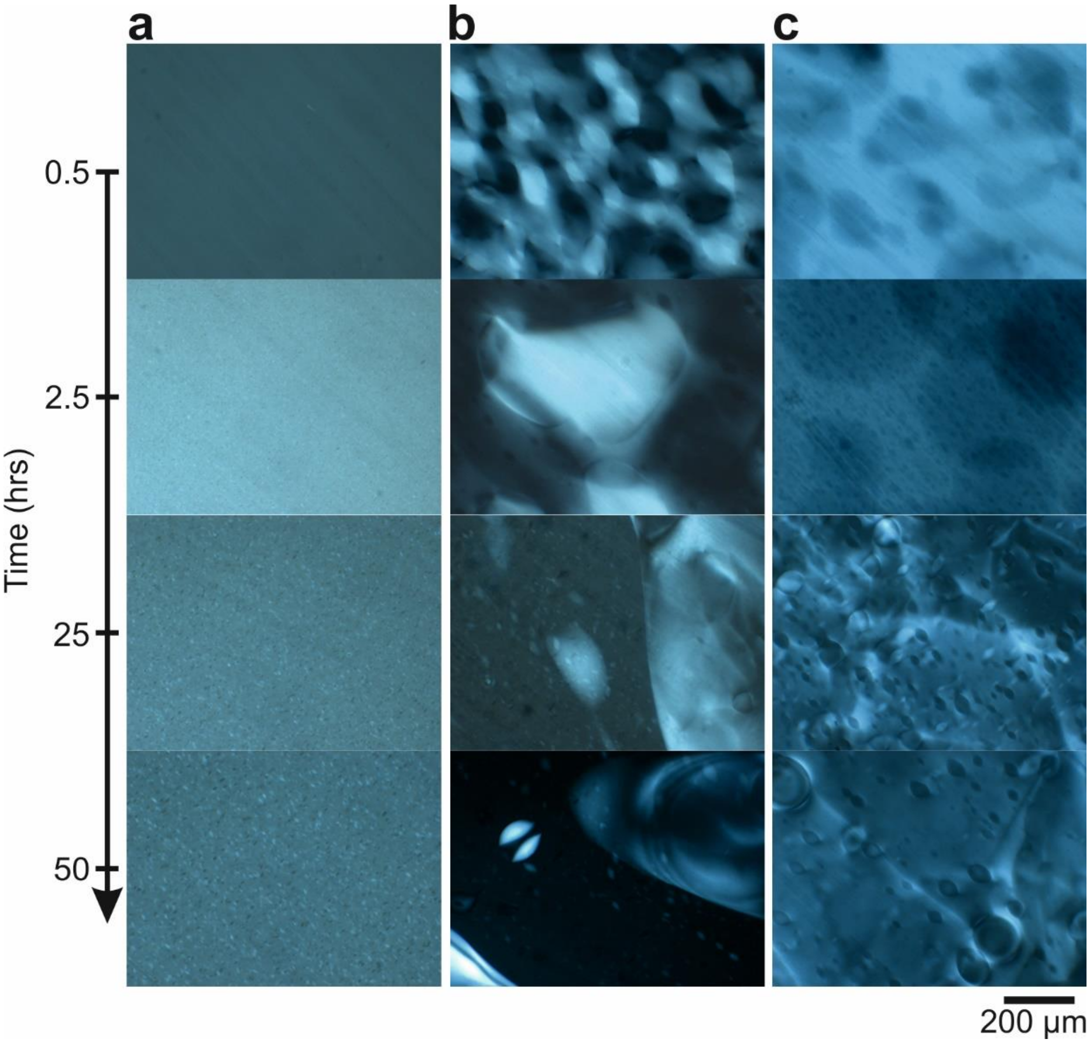

Supplementary Fig. 3 | Classical N&G time for the formation of tactoids. Three different sets of suspension of amyloid fibrils with concentrations, within Onsager branches (ϕ_{I} and ϕ_{N}), as: (**a**) just above ϕ_{I} at $1.008\phi_{\mathrm{I}}$, (**b**) midpoint of ϕ_{I} and ϕ_{N} at $(\phi_{\mathrm{I}} + \phi_{\mathrm{N}})/2$, and (**c**) just below ϕ_{N} at $0.996\phi_{\mathrm{N}}$. Within the time frame of observation, cholesteric tactoids are only observed in (**b**), but after two days. In the case of (**a**) mainly homogenous and a few bipolar tactoids are formed within the first two days. At the concentration corresponding to (**c**), mainly negative tactoids are formed with a few homogenous and bipolar tactoids that started to appear within 30 mins.

Supplementary Fig. 4

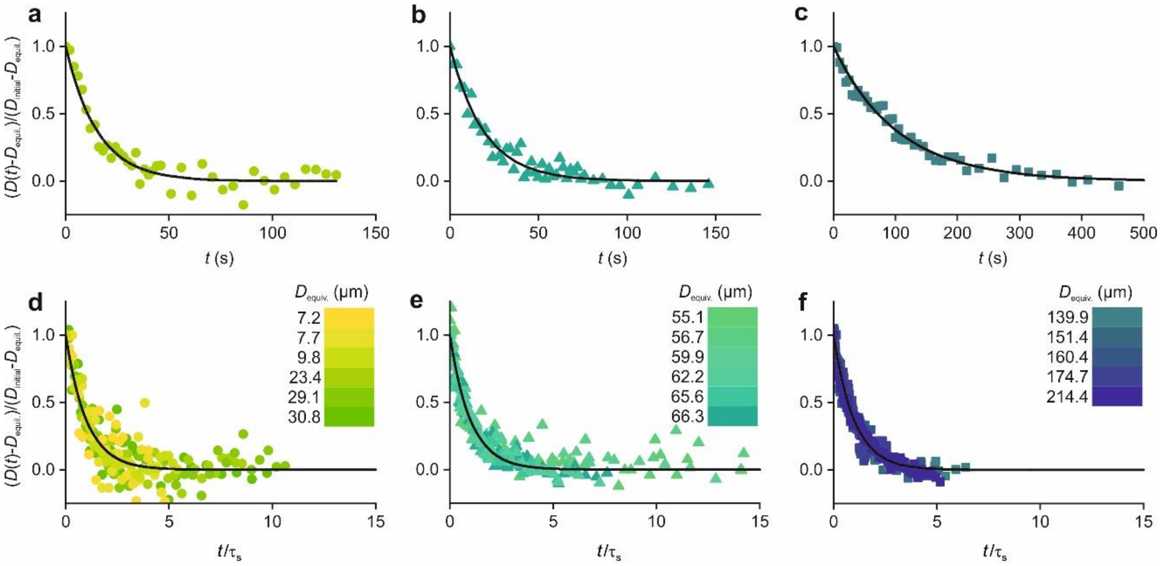

Supplementary Fig. 4 | Shape relaxation behaviour of the tactoids upon the breakup of the nematic jet. The filled circle, triangle, and square symbols illustrate homogenous, bipolar, and cholesteric tactoids, respectively. **a-c** $(D(t)\text{-}D_{equil.})/(D_{initial}\text{-}D_{equil.}) = \exp(-t/\tau_s)$, black lines, are fitted to obtain the characteristic shape relaxation time τ_s, see Ref. 43 in the main text for details on τ_s. **d-f** The results of tactoids of various internal structures, independent from the tactoids volume, follow a universal curve of $(D(t)\text{-}D_{equil.})/(D_{initial}\text{-}D_{equil.}) = \exp(-t/\tau_s)$, see Fig. 2c.

Supplementary Fig. 5

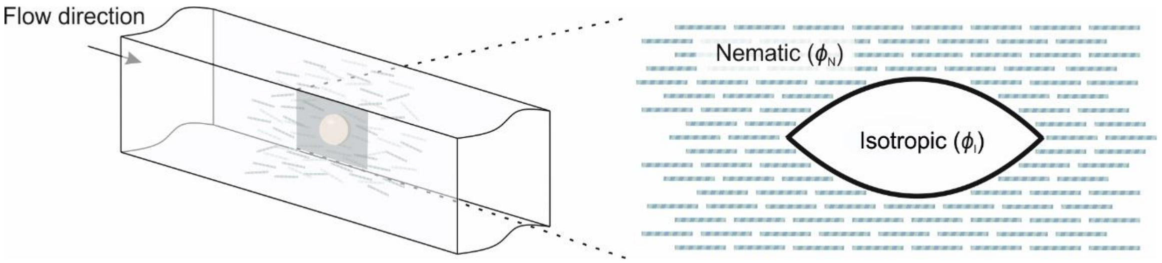

Supplementary Fig. 5 | Schematic of a negative tactoid in a nematic medium. Within the microfluidic channel, the fibrils in the nematic phase and tactoid are aligned along the flow direction.

Supplementary Fig. 6

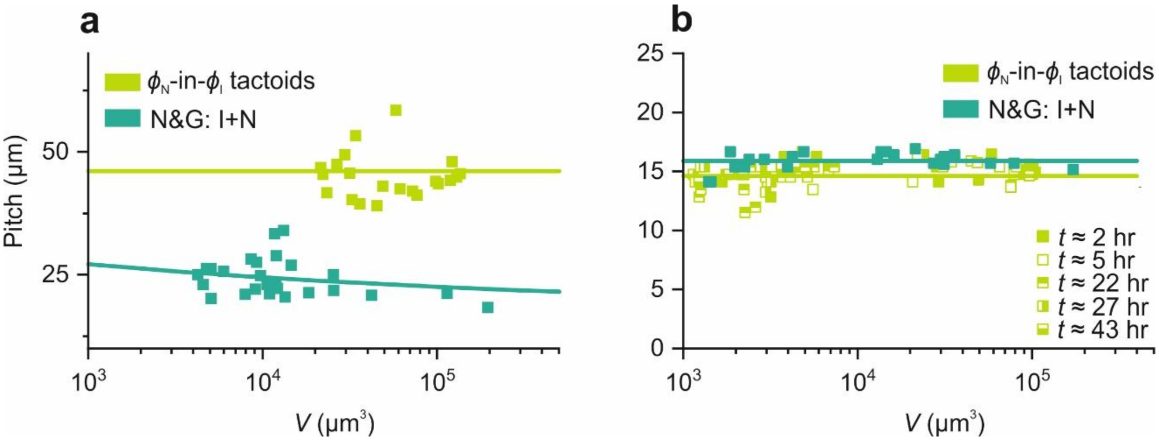

Supplementary Fig. 6 | Comparison of the pitch value of the cholesteric tactoids formed through N&G process and by extruding the Onsager branches (ϕ_N-in-ϕ_I tactoids). The pitch values are from the tactoids of amyloid fibrils (**a**) and cellulose nanocrystals (**b**). **a** While the pitch value decreases with an increase in volume for tactoids formed through phase separation via N&G, it stays constant at 46.1 μm for tactoids formed by extruding the Onsager branches. **b** Pitch stays constant for tactoids with various volumes formed by extruding the Onsager branches and via N&G pathways at 14.6 μm and 15.9 μm, respectively. Tracking the pitch of the tactoids over a long time shows no significant changes.

Supplementary Fig. 7

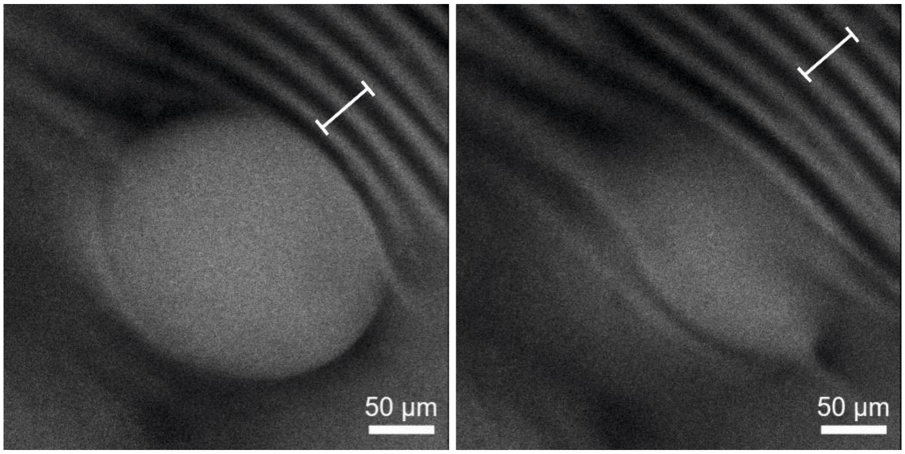

Supplementary Fig. 7 | The cholesteric pitch value measured from the cholesteric structure formed in the medium of the nematic phase with a concentration set at ϕ_N. To measure the true natural pitch in the bulk nematic phase, we injected the nematic phase set at ϕ_N as a medium phase (in the experiments of the inverse tactoids formation) and observed in the continuous phase the formation of the cholesteric structure with a pitch value of exactly 46.1 μm, highlighting again that 46.1 μm is the true equilibrium pitch.

Supplementary Note 1

To fully clarify how significant is the energy input from the flow field, in the following, we calculate both the thermodynamic energy of the system and the dissipative energy associated with the flow field which results in the formation of the interface through Rayleigh-Plateau instability. To start, we write the energy of hard rods freely rotating in the solution using the principle of equipartition of energy for a gas of rods having 3 degrees of freedom for the center of mass and one degree of freedom for angular positions (implying the rotation is polarized in a plane for conservation of angular momentum):

$$S_O = 4\left(\frac{1}{2}K_B T\right)cV \quad (1)$$

where the c, K_B, and T, the number density, Boltzmann constant, and temperature, respectively. We obtained the value of c as

$$c = \frac{N}{V} \quad (2)$$

where N stands for the number of colloids at a given volume V. We can obtain the value of the N as

$$N = \frac{\phi V}{\frac{1}{4}\pi {d_{\text{fibrils}}}^2 L_{\text{fibrils}}} \quad (3)$$

Therefore, from Eq. 2-3, one can see that

$$c = \frac{\phi}{\frac{1}{4}\pi {d_{\text{fibrils}}}^2 L_{\text{fibrils}}}. \quad (4)$$

By substituting Eq. 4 into Eq. 1, we have

$$S_O = 8\,\frac{K_B T\,\phi}{\pi {d_{\text{fibrils}}}^2 L_{\text{fibrils}}} V. \quad (5)$$

Where, by considering V as the volume of a jet with the length of L_{jet}, one can write

$$S_O = 2\,\frac{K_B T\ \phi}{{d_{\text{fibrils}}}^2 L_{\text{fibrils}}} {d_{\text{jet}}}^2 L_{\text{jet}}. \quad (6)$$

Eq. 6 gives the energy of a gas of hard rods. The excess free energy (deviation from perfect gas) has been calculated in detail by Onsager, which is in the order of 3 to 4 $*\ K_B TcV$ (Ref. 26 in the main text). This implies that the total thermodynamic energy of the system of interacting hard rods in the suspension will be, in any case, of the order of several $K_B TcV$.

Next, we calculate the energy given to the system, i.e. the energy that is required to create an extra surface when the stream breaks into droplets due to the extrusion and the Rayleigh-Plateau instability. The (additional) surface energy for such a breaking jet with the length of L_{jet} can be written as:

$$S_s = \pi\gamma\, d_{\text{jet}} L_{\text{jet}} \quad (7)$$

By substituting the values of the parameters in Eq. 6 (taking $2K_B TcV$ as a conservative case) and Eq. 7 as $K_B T$ =4.16×10^{-21}J mole^{-1}, γ =10^{-7} N m^{-1} , d_{fibrils}= 2.5 nm, L_{fibrils}= 303 nm, and d_{jet}= 48.3 µm (which is the diameter value for the jet leading to the formation of the cholesteric tactoids, see Supplementary Movie 7), we have $S_O = 2.0 \times 10^{-7}\, L_{\text{jet}}$ and $S_s = 1.5 \times 10^{-11}\, L_{\text{jet}}$. This shows that the dissipative energy is around four orders of magnitude smaller than the thermodynamic energy of the rod-like colloidal solution that we use.

Additionally, we can support the above argument even experimentally by comparing the experiments of the cellulose nanocrystals and amyloid fibrils in Fig. 1 showing that at similar flow conditions of the extrusion of the fibrils, we get different structures and dynamics for cellulose nanocrystals and amyloid fibrils, confirming that indeed the thermodynamic is the dominant factor (by several orders of magnitude).

Supplementary Note 2

Negative tactoids aspect ratio

To estimate the shape of the negative tactoids, we assume that the fibrils are oriented in the flow direction due to the flow-induced alignment. Thus, given that the negative tactoids also are oriented in the flow direction, we will have the system as illustrated in Supplementary Fig. 5. Note that this argument stands correct as the tactoids and fibrils move at a similar speed in the flow direction.

We consider the free energy landscape, where the total free energy F_E is described in the scaling form of Frank–Oseen elasticity theory as

$$F_E \sim \gamma R r[1 + \omega(r/R)^2] + KV(1/R)^2 + \frac{1}{2}K_2(n.\nabla \times n + q_\infty)^2 V, \quad (8)$$

where γ is the interfacial tension, ω is the anchoring strength, K is the Frank elastic constant for splay and bending (taken to be equal), K_2 is the Frank twist elastic constant, q (=2π/pitch) is the chiral wave, and n is the nematic director. Here, the first term represents the system's surface free energy, and the second and third terms represent bulk elastic free energy from splay+bend and twist, respectively. Since in the system illustrated in Fig. S5, there is no energy associated with bulk twist, splay, and bending, we can ignore the bulk elastic free energy and, given that $V = r^2R$, write the free energy as

$$F_E \sim \gamma V/r + \gamma\omega r^5/V, \quad (9)$$

next, to obtain the aspect ratio of the negative tactoids, we minimize the free energy as follows:

$$\partial F_E/\partial r \sim -\gamma V/r^2 + 5\gamma\omega r^4/V = 0, \quad (10)$$

this gives us

$$V^2/r^6 \sim 5\omega, \quad (11)$$

that can be rewritten as the following:

$$R/r \sim (5\omega)^{1/2}. \quad (12)$$

The above equation gives the aspect ratio of the negative tactoids, illustrating that it only depends on the anchoring strength. This confirms our experimental results in Fig. 3g that the aspect ratio of the tactoids is independent of the volume.
