# Supplementary material for: Disentangling kinetics from thermodynamics in heterogeneous colloidal systems
Source: Nat Commun. 2023 Feb 4;14:607. doi: 10.1038/s41467-023-36292-8 (PMC9899263; doi:10.1038/s41467-023-36292-8)
Supplement: Supplementary file 3 — Description of Additional Supplementary Files [file 41467_2023_36292_MOESM3_ESM.pdf]

Supplementary Movie 1 | Dynamics of the homogenous tactoids with $D_{\text{equiv.}}$ = 2.5 ± 0.2 µm formation. The jet diameter is 1.4 µm. The video was captured under crossed polarizers and the duration of the video is 1.15 min in real time. The white crossed arrows denote the crossed polarizers. The scale bar is 25 µm.

Supplementary Movie 2 | Dynamics of the homogenous tactoids with $D_{\text{equiv.}}$ = 4.2 ± 0.7 µm formation. The jet diameter is 2.3 µm. The video was captured under crossed polarizers and the duration of the video is 2.25 min in real time. The white crossed arrows denote the crossed polarizers. The scale bar is 100 µm.

Supplementary Movie 3 | Dynamics of the homogenous tactoids with $D_{\text{equiv.}}$ = 14.4 ± 1.6 µm formation. The jet diameter is 7.0 µm. The video was captured under crossed polarizers and the duration of the video is 5.33 min in real time. The white crossed arrows denote the crossed polarizers. The scale bar is 200 µm.

Supplementary Movie 4 | Dynamics of the bipolar tactoids with $D_{\text{equiv.}}$ = 28.5 ± 3.6 µm formation. The jet diameter is 12.3 µm. The video was captured under crossed polarizers and the duration of the video is 7.85 min in real time. The white crossed arrows denote the crossed polarizers. The scale bar is 200 µm.

Supplementary Movie 5 | Dynamics of the bipolar tactoids with $D_{\text{equiv.}}$ = 33.7 ± 4.9 µm formation. The jet diameter is 18.9 µm. The video was captured under crossed polarizers and the duration of the video is 7.85 min in real time. The white crossed arrows denote the crossed polarizers. The scale bar is 200 µm.

Supplementary Movie 6 | Dynamics of the cholesteric tactoids with $D_{\text{equiv.}}$ = 71.6 ± 7.8 µm formation. The jet diameter is 31.8 µm. The video was captured under crossed polarizers and the duration of the video is 17.78 min in real time. The white crossed arrows denote the crossed polarizers. The scale bar is 200 µm.

Supplementary Movie 7 | Dynamics of the cholesteric tactoids with $D_{\text{equiv.}}$ = 94.5 ± 7.3 µm formation. The jet diameter is 48.3 µm. The video was captured under crossed polarizers and the duration of the video is 31.66 min in real time. The white crossed arrows denote the crossed polarizers. The scale bar is 200 µm.

Supplementary Movie 8 | Dynamics of the negative tactoids formation. The jet diameter is 55.2 µm. The video is a sequence of 3D reconstructed images captured with confocal microscopy. The duration of the video (8.61 min in real time) and the scale bar are provided in the video.
